# Detection and classification of intracranial haemorrhage on CT images using a novel deep-learning algorithm

**DOI:** 10.1038/s41598-020-77441-z

**Published:** 2020-11-25

**Authors:** Ji Young Lee, Jong Soo Kim, Tae Yoon Kim, Young Soo Kim

**Affiliations:** 1 Department of Radiology, School of Medicine, Hanyang University Seoul Hospital, Seoul, Republic of Korea; 2 Institute for Software Convergence, Hanyang University, 222 Wangsimni-ro, Seongdong-gu, Seoul, 04763 Republic of Korea; 3 Department of Radiology, College of Medicine, Hanyang University Guri Hospital, Guri, Republic of Korea; 4 Department of Neurosurgery, School of Medicine, Hanyang University Seoul Hospital, 222 Wangsimni-ro, Seongdong-gu, Seoul, 04763 Republic of Korea

**Keywords:** Computational biology and bioinformatics, Medical research

## Abstract

A novel deep-learning algorithm for artificial neural networks (ANNs), completely different from the back-propagation method, was developed in a previous study. The purpose of this study was to assess the feasibility of using the algorithm for the detection of intracranial haemorrhage (ICH) and the classification of its subtypes, without employing the convolutional neural network (CNN). For the detection of ICH with the summation of all the computed tomography (CT) images for each case, the area under the ROC curve (AUC) was 0.859, and the sensitivity and the specificity were 78.0% and 80.0%, respectively. Regarding ICH localisation, CT images were divided into 10 subdivisions based on the intracranial height. With the subdivision of 41–50%, the best diagnostic performance for detecting ICH was obtained with AUC of 0.903, the sensitivity of 82.5%, and the specificity of 84.1%. For the classification of the ICH to subtypes, the accuracy rate for subarachnoid haemorrhage (SAH) was considerably excellent at 91.7%. This study revealed that our approach can greatly reduce the ICH diagnosis time in an actual emergency situation with a fairly good diagnostic performance.

## Introduction

Intracranial haemorrhage (ICH) is a critical medical emergency that requires rapid and prompt assessment and management^1,2^. Due to its high mortality rate (approximately 40%), early detection and classification on non-contrast computed tomography (CT) are essential for ensuring a favourable prognosis and limiting the occurrence of neurologic deficits^2–4^. However, an influx of CT scans tends to delay the early detection of ICHs due to a lack of prompt access to radiologists who read the scans, especially in academic institutions. Therefore, an automatic notification system using the deep-learning artificial intelligence (AI) method has been introduced for the detection of ICH^5^.

Recently, many attempts have been made to apply the deep-learning method for the detection of ICH on CT images^2,6,7^. This deep-learning method is a form of machine learning which uses multiple processing layers to learn representations of data with multiple levels of abstraction^8,9^. Previous studies using this method showed an excellent diagnostic performance for detecting ICH in individual CT images with the sensitivity and the specificity of 98% and 95%, respectively, similar to that of expert radiologists. In addition, the fully three-dimensional deep-learning approach (not on individual CT images) for detecting ICH was reported^6^. All these studies used the back-propagation method^10–12^ for the learning algorithm and the convolutional neural network (CNN), which has self-organisation and pattern recognition abilities without human programming. Accordingly, this approach is a generic and problem agnostic method, not a rule-based and problem specific method^13^. However, it remains difficult to explain how this method generates the results from the input data^14^.

Previously, we (J. S. Kim) reported a novel deep-learning algorithm for artificial neural networks (ANNs): “Kim-Monte Carlo algorithm”^15^ for predicting the location of the glottis in video laryngoscopy images. The novel deep-learning algorithm was based on the Monte Carlo simulation, which is similar to the biological evolution of animals with a trial-and-error process^15^, but not an unnaturally mathematical procedure, i.e., the back-propagation method^10–12^ which has been the most commonly used method to date for training ANNs. An ANN includes hundreds of thousands or more unknown variables, i.e., weight factors and bias values. For the back-propagation method, each weight factor is adjusted by calculating the delta-value with employing the gradient descent method using all or a part of the learning data, which is carried out individually for all the hundreds of thousands or more weight factors in the backward direction, repeatedly until the training session is finished^15^. Therefore, the back-propagation method takes massive computing resources for training an ANN even with not a large amount of learning data. For the novel deep-learning algorithm^15^, randomly selected weight factors and bias values are adjusted by the amounts of randomly picked delta-values within a given range, not by calculating the delta-values using the computing-intensive gradient descent method, where the average training error for all the learning data of the current ANN is calculated repeatedly in the forward direction only^15^. Consequently, the algorithm performs a random optimisation process during the training session, which determines the weight factors and bias values of the ANN, minimising the training error for learning data. Thus, the algorithm^15^ is intuitively understandable, simple, and efficient.

There have been no studies to evaluate the diagnostic performance of the novel deep-learning algorithm in emergency neuroradiology. Therefore, the purpose of this study was to investigate the diagnostic performances of the algorithm^15^ without employing CNN, which is the most widely used deep-learning method currently for image recognition, for detecting ICH and for classifying the ICH into three subtypes, i.e., epidural haemorrhage (EDH)/subdural haemorrhage (SDH), subarachnoid haemorrhage (SAH), and intraparenchymal haemorrhage (IPH)/intraventricular haemorrhage (IVH).

In this study, CT images were divided into 10 subdivisions based on the intracranial height, and the CT images of a subdivision were summed into one image for each case. Then, the diagnostic performances for detecting ICH and for classifying the ICH into three subtypes were evaluated for each subdivision of CT images, and this study was the first of its kind. To compare the diagnostic performance of our approach with that of other approaches using CNN, all the CT images of each case were summed into one image regardless of the height of the intracranial part, and were also experimented. The results were compared with that of the fully three-dimensional deep-learning approach^6^ using CNN.

## Methods

### Subjects

The institutional review board (IRB) of Hanyang University Seoul Hospital (Seoul, Republic of Korea) approved this study, and confirmed that all methods in this study were performed in accordance with the Good Clinical Practice guidelines with the need for informed consent waived (IRB No. HYUH 2020-04-001).

All subjects underwent non-contrast brain CT between December 2017 and March 2019. The subjects (total 250 cases with 9085 CT images) included 100 normal cases and 150 ICH cases. The 250 patients were randomly divided into 166 for the training set and 84 for the validation set**.** The training set consisted of 66 normal cases and 100 ICH cases: 31 EDH/SDHs, 29 SAHs, and 40 IPH/IVHs; while the validation set consisted of 34 normal cases and 50 ICH cases: 13 EDH/SDHs, 16 SAHs, and 21 IPH/IVHs. Because the incidence of IVH was low, we included IVH into the type 3, i.e., IPH with or without IVH.

### Preparing image data

The CT scans were performed using SOMATOM Definition Edge (Siemens Healthcare, Erlangen, Germany). The parameters for non-contrast CT were as follows: 100 kVp, 200 mAs, and 5-mm slice thickness.

Two board-certified neuroradiologists (J. Y. Lee and T. Y. Kim) independently performed the image analysis for the presence of ICH and their classification. Finally, they performed the image analysis for the discordant cases in consensus. The images were reviewed with the following brain window setting: window width of 90 HU and window level of 40 HU. We used the review results as the learning data to train various ANNs and as a reference for comparison with the detection and classification results of ANNs.

Regarding data augmentation, we applied horizontal image flipping to the training set to double the number of training data^16^. Thus, the training set was doubled to 332 cases consisting of 132 normal cases and 200 ICH cases: 62 EDH/SDHs, 58 SAHs, and 80 IPH/IVHs.

**Figure 1:**
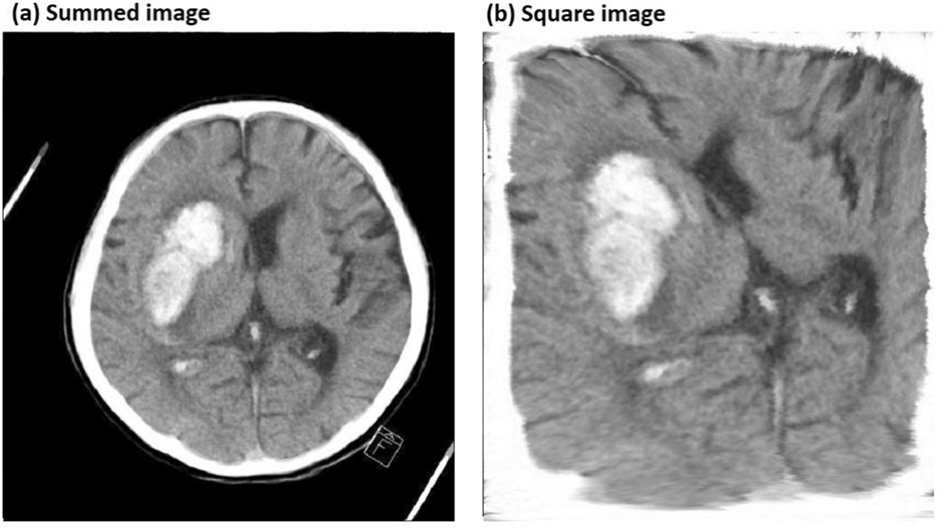
Example of CT image processing for preparing image data. (**a**) Summed image of a subdivision for a case. (**b**) Square image expanded to fit in a square.

For the localisation of ICH, the CT images were divided into 10 subdivisions based on the intracranial height, where each subdivision comprised 3–4 CT brain slices for a case. For each case, the CT images of a subdivision were summed into one image (Fig. 1a), and then expanded to fit the square image (Fig. 1b), to eliminate the differences in size and tilt of the skull image among the cases.

Of the 10 subdivisions based on the intracranial height, the subdivisions with relative heights of 0–10%, 11–20%, 81–90%, and 91–100% were excluded from this study since they have a very low incidence of ICH. Therefore, various ANNs were applied to the six subdivisions with relative heights of 21–30%, 31–40%, 41–50%, 51–60%, 61–70%, and 71–80% to detect ICH and to classify the ICH into its subtypes.

**Figure 2:**
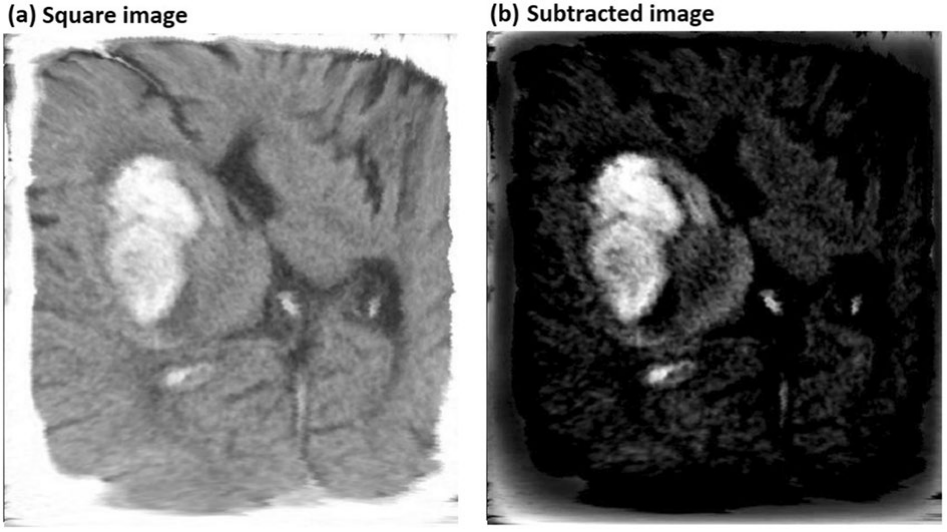
Example of CT image processing for preparing image data. (**a**) Square image expanded to fit in a square. (**b**) Subtracted image to eliminate the skull image and other images not related to ICH.

To classify the ICH into subtypes, only the CT images (or the cases) with ICH were applied for the training set (total 200 cases) and validation set (total 50 cases). From the square images (Fig. 2a) with ICH of each subdivision, the average square image of the CT images with normal findings included in the training set of the corresponding subdivision was subtracted (Fig. 2b), to eliminate the skull image and others not related to ICH. Then, various ANNs were applied to the subtracted square images as the training set and the validation set, to classify the ICH into subtypes. We classified ICH into three subtypes: EDH/SDH as type 1, SAH as type 2, and IPH/IVH as type 3.

Meanwhile, to compare the diagnostic performance of the novel deep-learning algorithm^15^ without employing CNN for detecting ICH, with that of the completely three-dimensional deep-learning approach^6^ (not to individual images) using the back-propagation method^10–12^ and CNN, all the CT images of each case were summed into one image regardless of the height of the intracranial part, and were also applied to various ANNs.

**Figure 3:**
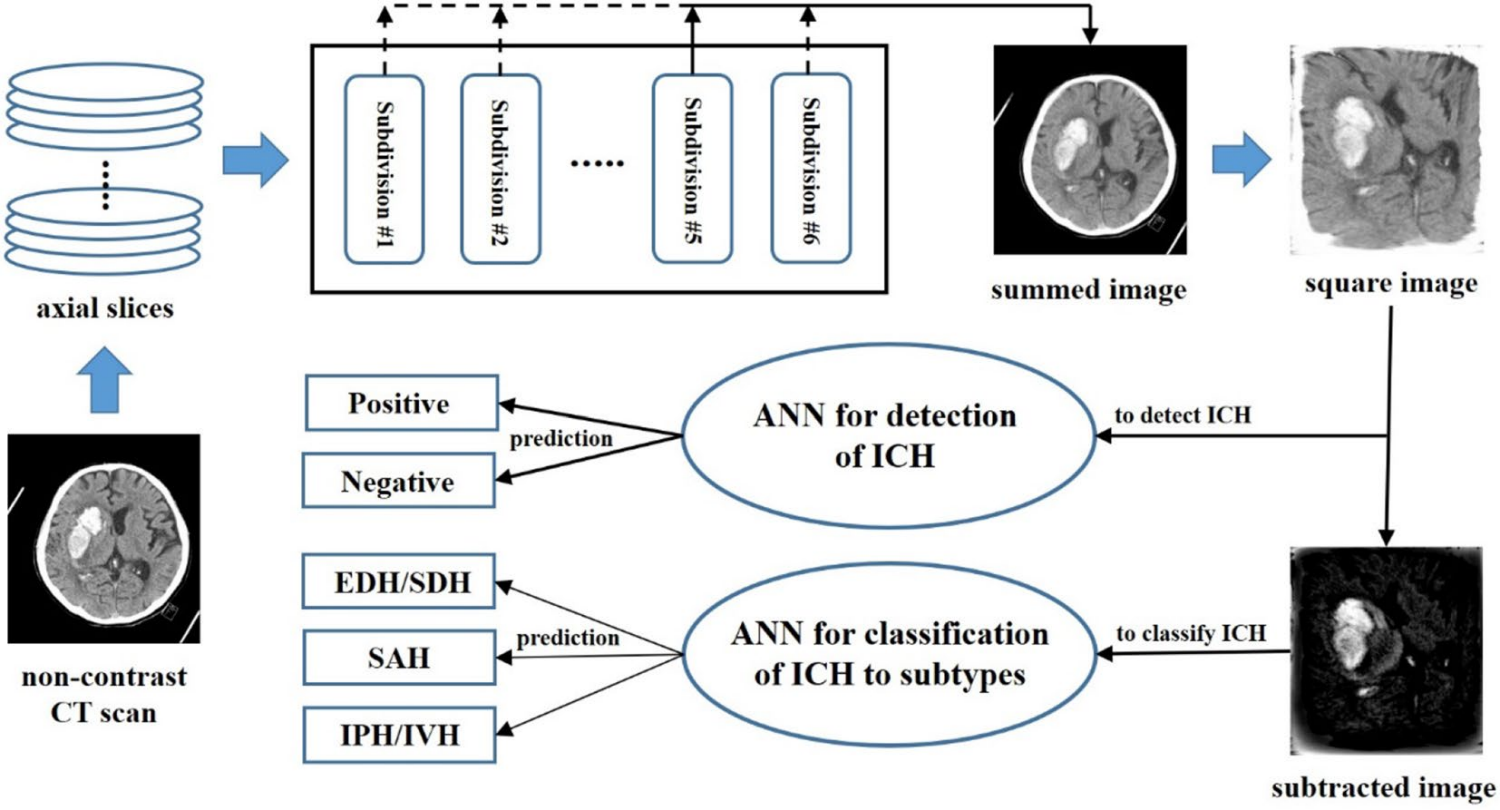
Schematic representation of the pipeline to detect and classify ICH on brain CT.

Figure 3 shows a schematic representation of the pipeline for detecting ICH and classifying the ICH into subtypes.

### Structures of ANN models

To find an extremely small ANN structure applying the novel deep-learning algorithm, a simple training process, that has a diagnostic performance comparable to that of a state-of-the-art deep-learning technology such as a CNN, the input image resolution and the number of hidden layers of an ANN model started at 16 × 16 and 1, respectively, and were gradually increased.

The resolutions of the square images (see Figs. 1b and 2b) of the training and validation sets were reduced to 16 × 16, 20 × 20, 24 × 24, 28 × 28, 30 × 30, 40 × 40, and 80 × 80 pixels. The number of input nodes of an ANN should be the total number of pixels of the square image with a reduced resolution. Thus, each pixel was converted to the black-and-white colour and its colour value was divided by the maximum value (255), to convert it to a value between 0.0 and 1.0, to be the input value of an input node^15^. Thus, in this study, CNN was not used to prepare the input data for ANNs.

The number of output nodes of an ANN should be 2 and 3 for detecting ICH and for classifying the ICH into subtypes, respectively. For the ANN models to detect ICH, the target values of the first output node (the positive node) and second output node (the negative node) were set at 1 and 0, respectively, for the CT images with ICH and vice versa. The output value of an ANN to detect ICH for a given input data was obtained using the following formula:

1 $$value = \, \left( {output[p]{-}output[n] + { 1}} \right) \, /{ 2,}$$

where *output[p]* and *output[n]* denote the output values of the positive node and the negative node of an ANN, respectively. The output values of an ANN to detect ICH were used for the receiver operating characteristic (ROC) analysis. For the ANN to classify the ICH into subtypes, the target values of three output nodes were set to [1, 0, 0] for type 1, [0, 1, 0] for type 2, and [0, 0, 1] for type 3.

The number of hidden layers of an ANN was set to 1, 2, or 3. The number of intermediate nodes in a hidden layer was changed from 10 to 240. Accordingly, various small ANN models, i.e., 120 to 140 models, were constructed for each of the six subdivisions.

### ANN training process

The novel deep-learning algorithm, “Kim-Monte Carlo algorithm,”^15^ a simple ANN training process, that differed from the back-propagation method^10–12^ was applied to train the various small ANN models with the training set as learning data. The initial weight factors and bias values of an ANN were randomly chosen within the range of − 0.2 to + 0.2^15^. The randomly selected weight factors and bias values of the ANN were repeatedly adjusted by the amounts of the randomly picked delta-values within the range of − 0.1 to + 0.1^15^, not using the gradient descent as does the back-propagation method^10–12^. Accepting or rejecting the adjustments was depending on whether or not the new values decrease the average training error of the ANN for all training data^15^, and 30 attempts were made to adjust the values of the selected variables in small random amounts. During a training cycle, the total sum of the randomly selecting ratio of variables was set to 900% of the total number of variables in the ANN. A training session was terminated after 10 repetitions of the training cycle, during which the randomly selecting ratio of the variables of a training cycle was steadily decreased from 15% to 1.5%^15^. After the training session of the ANN with the training set, the validation set was applied to the ANN to obtain the validation results, including the output values calculated using Eq. (1), to identify the diagnostic performance of the ANN model.

**Figure 4:**
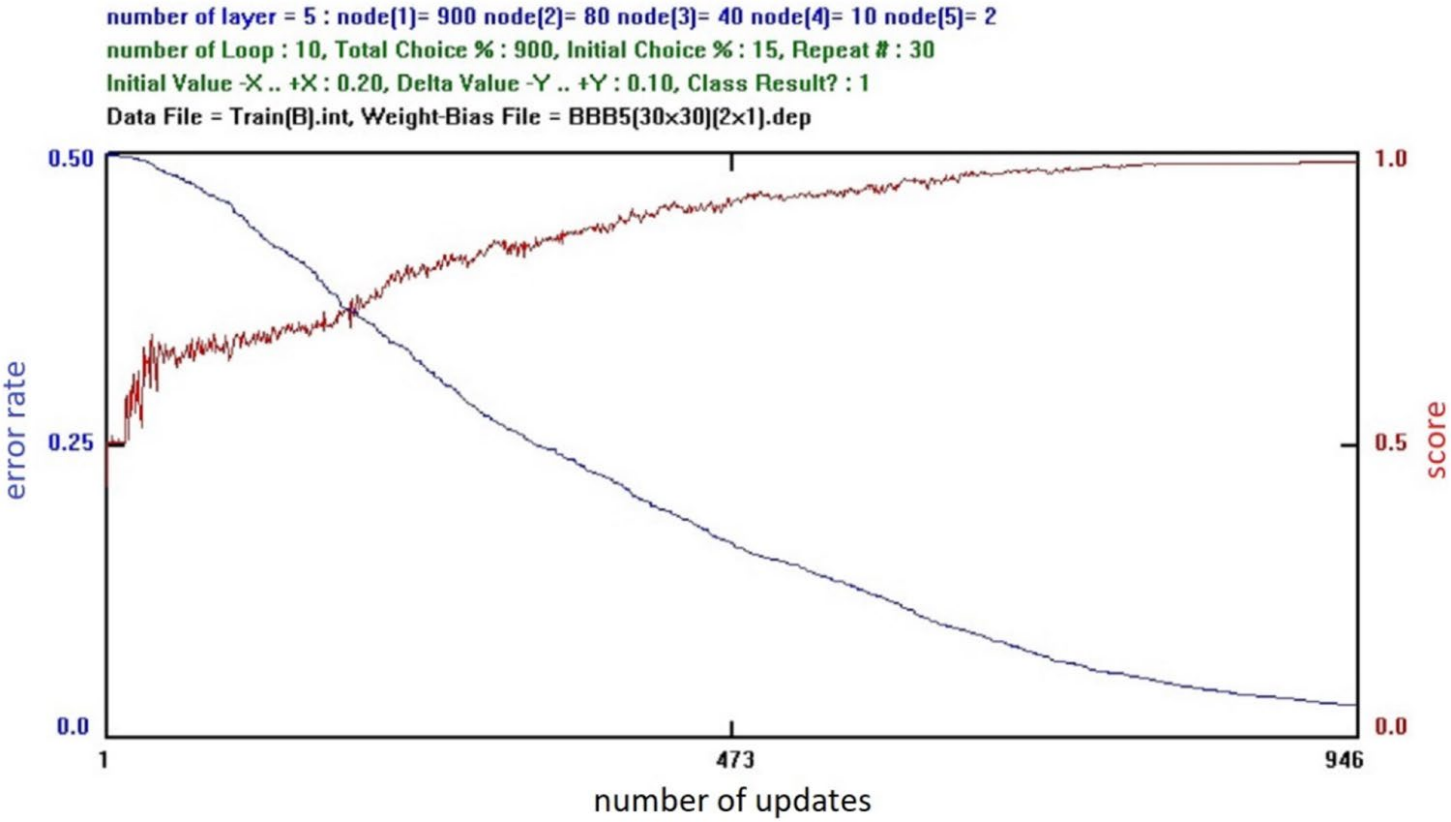
Computer screen for the training progress of an artificial neural network.

Figure 4 shows a computer screen for an ANN training progress. The blue curve denotes the ‘‘error rate’’ which is the average value of the training errors for all the training data set, where the training error is the summation of all the output nodes of the square of the difference between the output value of an output node and the corresponding target value for a given input data^15^. Thus, ‘‘error rate’’ is the absolute criterion of the training in progress. The horizontal value indicates the number of successfully changing values of the weight factors and the bias values of the ANN during the training session^15^. The red curve denotes the ‘‘score’’ which is the average score for all the training data set, where the score for a given input data is 1.0, if the output node with the maximum output value corresponds to the output node with the maximum target value, and 0.0 for other cases^15^. For detecting ICH, the ‘‘score’’ was the same with the accuracy that the cut-off value was set to 0.5 arbitrarily for the ROC curve analysis [refer to Eq. (1)]. Thus, the ‘‘score’’ is a subsidiary reference value of the training in progress^15^.

### Statistical analysis

We performed the ROC curve analysis to determine the diagnostic performances of various ANNs for the detection of ICH. Statistical analyses were conducted using commercially available software (SPSS, version 18 for Windows, SPSS, Chicago, IL, USA).

## Results

### ICH detection

To assess the diagnostic performance of our approach for the detection of ICH comparing with that of the completely three-dimensional deep-learning approach^6^, all the CT images of each case were summed into one image regardless of the height of the intracranial part, for the training set and the validation set, and the summed images were applied to train 90 ANN models. The ANN structure comprised 400 input nodes (for the resolution of 20 × 20 pixels), 40 intermediate nodes in the first hidden layer, 20 intermediate nodes in the second hidden layer, 10 intermediate nodes in the third hidden layer, and two output nodes, was chosen by comparing the validation results among the 90 ANN models. Using the ANN model, we obtained that AUC was 0.859, and the sensitivity and the specificity were 78.0% and 80.0%, respectively.

Table 1 presents the ICH detection results with the validation set for the six subdivisions with relative heights of 21–30%, 31–40%, 41–50%, 51–60%, 61–70%, and 71–80%, where the ANN model for a given subdivision was chosen by comparing the validation results among 60–70 models. The accuracy in Table 1 was obtained using the following formula:

2 $$accuracy = \, \left( {N[p] \times {\text{ sensitivity }} + N[n] \times {\text{ specificity}}} \right) \, /{ 84,}$$

where *N[p]* and *N[n]* denote the numbers of positive cases and negative cases in the validation set, respectively, and 84 indicates the total number of cases in the validation set, i.e., *N[p]* + *N[n].*

**Table 1:** ICH detection results using the validation set for the six subdivisions.

Subdivision	Resolution	Positive case	Negative case	Hidden nodes	AUC	Sensitivity %	Specificity %	Accuracy %
21–30%	28 × 28	12	72	40	0.838	91.7	70.8	73.8
31–40%	24 × 24	27	57	80	0.870	92.6	73.7	79.8
41–50%	30 × 30	40	44	40	0.903	82.5	84.1	83.3
51–60%	80 × 80	37	47	120	0.845	70.3	87.2	79.8
61–70%	30 × 30	31	53	240	0.764	83.9	69.8	75.0
71–80%	30 × 30	21	63	40	0.825	81.0	71.4	73.8
Average	–	–	–	–	0.841	83.7	76.2	77.6

Among the six subdivisions, we obtained the best ICH detection results in the subdivision with relative height of 41–50%, which had the most number of positive cases, and AUC and the accuracy were 0.903 and 83.3%, respectively.

### ICH classification

Table 2 reports the results of ICH classification into subtypes, with the validation set for the six subdivisions with relative heights of 21–30%, 31–40%, 41–50%, 51–60%, 61–70%, and 71–80%, where the ANN model for a given subdivision was chosen by comparing the validation results among 60 to 70 models.

**Table 2:** Classification results of the ICH into subtypes using the validation set for the six subdivisions.

Subdivision	Reolution	Hidden nodes	Type 1		Type 2		Type 3		Accuracy %
			predicted #	Case #	predicted #	Case #	predicted #	Case #	
21–30%	24 × 24	80–40-10	0	1	9	9	1	2	83.3
31–40%	24 × 24	20–10	3	6	12	13	3	8	66.7
41–50%	80 × 80	120–30	7	9	13	15	9	16	72.5
51–60%	28 × 28	80–40–10	3	8	10	12	13	17	70.3
61–70%	30 × 30	80–40–10	5	10	6	6	10	15	67.7
71–80%	30 × 30	40–20–10	5	9	5	5	3	7	61.9
Total	–	–	23	43	55	60	39	65	69.6

Table 2 shows that the best classification results among the three subtypes were obtained the accuracy rate of 91.7% (55/60) for type 2 (SAH), compared to the overall accuracy rate of 69.6%.

## Discussion

We applied the novel deep-learning algorithm^15^ to detect and classify ICH on brain CTs with small datasets. Since our approach was not CNN-based deep-learning method, data selection and preprocessing of the input images for ANNs were not essential. For the detection of ICH, AUC was 0.903 for CT images of the subdivision with relative height of 41–50%. For the classification of the ICH into subtypes, although the overall accuracy rate was 69.6%, the accuracy rate for type 2 (SAH) was the highest at 91.7%. Therefore, this study revealed that our approach has a potential for clinical application as an alternative in neuroradiology emergency.

Recently, the fully three-dimensional deep-learning approach using CNN for detecting ICH was published^6^. In their study, the researchers developed this architecture and used it to detect ICH in head CTs. They reported that AUC was 0.846, and the sensitivity was 73.0% when the specificity was chosen to 80.0%. Applied to the summation of all CT images for each case regardless of the height of the intracranial part, we obtained that AUC was 0.859, and the sensitivity and the specificity were 78.0% and 80.0%, respectively. Therefore, the diagnostic performance of our approach for detecting ICH was compared favorably with that of other approaches using CNN.

There have been studies that applied the deep-learning method to detect ICH on individual CT images using the back-propagation method^10–12^ and CNN, unlike this study which detected ICH on summed CT images using the novel deep-learning algorithm^15^ without using CNN, as well as the three-dimensional approach^6^. A study with customised, region-of-interest (ROI)-based CNN for haemorrhage detection showed a higher AUC of 0.983 with the sensitivity and the specificity of 97.1% and 97.5%^2^, respectively. Another study for detecting haemorrhage, mass effect, and hydrocephalus showed AUC of 0.91, and the sensitivity and the specificity of 90.0% and 85.0%^5^, respectively. While these studies achieved very high diagnostic performances for individual images, there is a limitation to their practical application in general emergency situations. The application may be dependent on the type of CT scanner used, and data selection and preprocessing for the input CT images may be essential. In this study, we obtained comparable performance results using small datasets^2,5–7,14,17^. In addition, our approach has the advantage that the preprocessing step requiring an expert can be completely omitted, such that the entire process can be fully automated and embedded into the CT scanner. Therefore, our approach can greatly reduce the ICH diagnosis time in an actual emergency setting without the help of a specialist.

For the classification of the ICH into subtypes, the accuracy rates for type 1 (EDH/SDH) and type 3 (IPH/IVH) were lower than that for type 2 (SAH). We presumed that this might have resulted from the small amount of haemorrhage. Additionally, in cases of EDH/SDH, the density of the haemorrhage could be similar to that of the skull; hence, these small amounts of EDH/SDH might have been missed. Therefore, further studies with the subdural window setting or with skull removal are needed to enhance the accuracy rate of the ICH classification.

There are some limitations to this study. First, the sample size was small. Further studies with large datasets will be needed for the validation of this approach. Second, we performed expanding the images to fit in a square to eliminate inter-individual variance of the brain size and scanned angle. It might have distorted the original shape of haemorrhage on the CT image. Third, we did not include CT images with surgical devices such as clips or VP shunts and from patients with postoperative states such as craniectomy. Due to the fact that these conditions could influence the results, further studies are needed to validate our approach for clinical applications.

In conclusion, the novel deep-learning algorithm for ANNs was experimentally tested to detect and classify ICH using small datasets; it showed a fairly good diagnostic performance. Our approach has the advantage of being fully automated, having a fast processing time due to omission of the preprocessing step, and having the ability to be embedded into the CT scanner. For clinical application, further methodological development and validation studies with large datasets are required.

## Data Availability

The datasets generated and/or analysed during the current study are not publicly available due to the confidential nature of the clinical imaging data and the potential risk of personal information leakage, but can be made available by the corresponding authors on reasonable request.
